# Detecting selected haplotype blocks in evolve and resequence experiments

**DOI:** 10.1111/1755-0998.13244

**Published:** 2020-09-06

**Authors:** Kathrin A. Otte, Christian Schlötterer

**Affiliations:** ^1^ Institut für Populationsgenetik Vetmeduni Vienna Vienna Austria

**Keywords:** data‐driven parameter choices, evolve and resequence, experimental evolution, haplotype reconstruction, replicated time series data, selection, sequencing of pooled individuals

## Abstract

Shifting from the analysis of single nucleotide polymorphisms to the reconstruction of selected haplotypes greatly facilitates the interpretation of evolve and resequence (E&R) experiments. Merging highly correlated hitchhiker SNPs into haplotype blocks reduces thousands of candidates to few selected regions. Current methods of haplotype reconstruction from Pool‐seq data need a variety of data‐specific parameters that are typically defined ad hoc and require haplotype sequences for validation. Here, we introduce haplovalidate, a tool which detects selected haplotypes in Pool‐seq time series data without the need for sequenced haplotypes. Haplovalidate makes data‐driven choices of two key parameters for the clustering procedure, the minimum correlation between SNPs constituting a cluster and the window size. Applying haplovalidate to simulated E&R data reliably detects selected haplotype blocks with low false discovery rates. Importantly, our analyses identified a restriction of the haplotype block‐based approach to describe the genomic architecture of adaptation. We detected a substantial fraction of haplotypes containing multiple selection targets. These blocks were considered as one region of selection and therefore led to underestimation of the number of selection targets. We demonstrate that the separate analysis of earlier time points can significantly increase the separation of selection targets into individual haplotype blocks. We conclude that the analysis of selected haplotype blocks has great potential for the characterization of the adaptive architecture with E&R experiments.

## 1 INTRODUCTION

Experimental evolution combined with whole‐genome resequencing (E&R) is an effective approach to detect genomic signatures of adaptation (Turner, Miller, & Cochrane, 2013). E&R studies on complex sexually reproducing organisms like *Drosophila* use polymorphic founder populations, and selection acts mainly on standing genetic variation instead of new mutations (Tenaillon et al., 2012). Here, the power to detect selected alleles significantly increases with an increasing number of replicates (Kofler & Schlötterer, 2014; Long, Liti, Luptak, & Tenaillon, 2015) and time points (Burke, Liti, & Long, 2014), and the most economic approach is to sequence pools of individuals (Pool‐seq) instead of individual genomes (Schlötterer, Tobler, Kofler, & Nolte, 2014). While estimating population allele frequencies accurately, Pool‐seq does not provide linkage information. Therefore, E&R studies typically treat individual SNPs as independent instead of incorporating the underlying haplotype structure, and frequently report an excess of outlier SNPs responding to selection (Burke et al., 2010; Griffin, Hangartner, Fournier‐Level, & Hoffmann, 2017; Jha et al., 2015; Orozco‐terWengel et al., 2012; Remolina, Chang, & Leips, 2012; Tobler et al., 2014; Turner et al., 2013; Turner, Stewart, Fields, Rice, & Tarone, 2011), which is not compatible with population genetic theory (Nuzhdin & Turner, 2013).

Franssen, Nolte, Tobler, and Schlötterer (2015) shed some light on the excess of candidate loci by jointly analysing Pool‐seq data and experimentally phased haplotypes from the same experiment. They pointed out that a high number of the candidate SNPs in *Drosophila melanogaster* studies were either located in large segregating inversions (Kapun, Van Schalkwyk, McAllister, Flatt, & Schlötterer, 2014) which suppress recombination or in genomic regions with reduced recombination rates. Another factor contributing to the large number of candidate SNPs is selection on low‐frequency alleles. The moderate number of recombination events during the experiment is not sufficient to break up the association between the target of selection and linked neutral variants that were private to the selected low‐frequency haplotype. These results show that understanding the genomic architecture of adaptation is a very challenging task and individual haplotypes from evolved populations greatly facilitate it by providing linkage information.

Sequencing of individuals in combination with phasing is a widely used approach to infer haplotype data (Browning & Browning, 2007; Delaneau, Howie, Cox, Zagury, & Marchini, 2013; Delaneau Zagury & Marchini, 2013; Li, Willer, Ding, Scheet, & Abecasis, 2010; Marchini, Howie, Myers, McVean, & Donnelly, 2007; Stephens, Smith, & Donnelly, 2001). However, in populations with low‐linkage disequilibrium, statistical phasing of these individual genomes is still a challenge (Bukowicki, Franssen, & Schlötterer, 2016). Alternatively, phased haplotypes can be generated experimentally, but this method requires living organisms (Franssen et al., 2015; Langley, Crepeau, Cardeno, Corbett‐Detig, & Stevens, 2011). An alternative approach is the statistical inference of haplotypes from Pool‐seq data. Taking advantage of sequenced founder haplotypes, the haplotypes of evolved individuals have been determined by regression (Long et al., 2011), a hidden Markov model (Cubillos et al., 2013), maximum likelihood (Kessner, Turner, & Novembre, 2013) and a system of linear equations (Cao & Sun, 2015). These methods rely on the complete knowledge of all involved founder haplotypes (Cubillos et al., 2013) and are limited to a restricted window size because otherwise the error rate is too high (Cao & Sun, 2015; Kessner et al., 2013; Long et al., 2011).

A different approach to reconstruct selected haplotype blocks without information about the founder haplotypes was proposed by Franssen, Barton, and Schlötterer (2017). This approach uses window‐based correlation analysis of allele frequency data across replicates and time points combined with hierarchical clustering. Each cluster of SNPs corresponds to a selected haplotype block. Franssen, Barton, et al. (2017) focused on haplotype blocks starting from low allele frequencies (≤0.03), and marker SNPs (i.e. correlated SNPs increasing in frequency), which are mostly private to them, can be identified by strongly correlated allele frequency changes. This approach successfully identified selected haplotype blocks up to several Mb in simulated and empirical Pool‐seq data. Extending the approach of Franssen, Barton, et al. (2017) to haplotypes by including also alleles with higher starting frequencies, Barghi et al. (2019) successfully reduced over 50,000 outlier SNPs to 99 reconstructed haplotype blocks responding to selection in experimentally evolved *Drosophila simulans* populations. Both, Barghi et al. (2019) and Franssen, Barton, et al. (2017) relied on experimentally phased haplotypes of evolved populations to validate their results. Without sequences of evolved and ancestral haplotypes, the validation of reconstructed blocks is challenging, as haplotype reconstruction requires ad hoc choices of key parameters which can change the outcome dramatically and are highly dependent on the data set.

Here, we propose a new approach to define the haplotype reconstruction criteria to detect independent genomic regions with selection signatures. It is suitable for most E&R experiments, avoids ad hoc choices of clustering parameters and does not depend on the availability of phased haplotype data. Our approach takes advantage of the full genomic data to distinguish between statistically significant clustering, most likely caused by directional selection, and random associations. It is implemented in the r package haplovalidate.

## 2 MATERIALS AND METHODS

If not stated otherwise, all analyses were conducted with r version 3.6.0 (R Core Team, 2019) and the r package poolseq version 0.3.1 (Taus, Futschik, & Schlötterer, 2017) and haploReconstruct 0.1.3_3 (Franssen, Barton, et al., 2017).

### 2.1 Haplotype reconstruction

**FIGURE 1:**
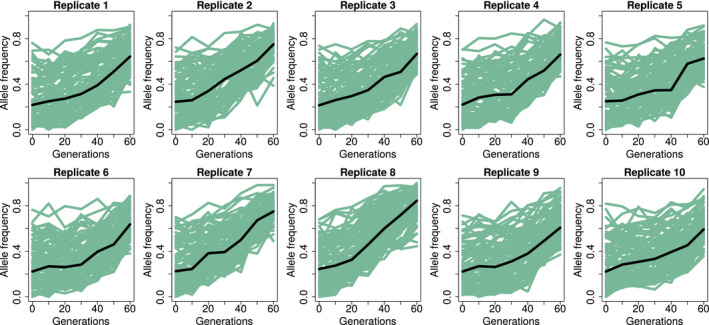
Example of correlated allele frequency trajectories in a reconstructed haplotype block (simulated data). The simulations were performed over 60 generations (*x*‐axis) and allele frequencies (*y*‐axis) were detected in 10 replicates. Each green line is a haplotype block marker SNP, and each box is a different population replicate. The black line is the median for all haplotype block marker SNPs in a replicate [Colour figure can be viewed at wileyonlinelibrary.com]

The haplotype reconstruction approach applied by haplovalidate was proposed by Franssen, Barton, et al. (2017) and implemented in the r package haploReconstruct. It is based on the idea that SNPs on the same haplotype block should behave similarly over time, that is, have highly correlated allele frequency trajectories. The boundaries of haplotype blocks on the chromosomes are formed by the interplay of recombination and evolutionary forces. Selected haplotype blocks increasing in frequency can be monitored by following the trajectories of their marker SNPs. This approach is especially suitable for E&R experiments, as multiple time points and population replicates increase the power of detecting correlated signals (for an example see Figure 1). Franssen, Barton, et al. (2017) proposed to analyse the correlation of SNPs across replicates and time points within overlapping genomic windows and apply hierarchical clustering. Different groups are then separated by applying a correlation cut‐off, with each cluster of SNPs corresponding to a reconstructed haplotype block.

### 2.2 Haplovalidate

The results of an E&R experiment depend greatly on the underlying adaptive architecture, for example selection scenario, number of selected loci, initial starting frequencies of selected alleles, strength of selection and linkage structure of the founder population. One selected haplotype block can consist of a group of haplotypes with some sequence variation, but all haplotypes are sharing the same selected allele(s). As a consequence, the correlation of SNPs in the haplotype block depends on the extent they are shared among selected haplotypes and nonselected ones. This results in variation of the correlation of shared marker SNPs within one selected haplotype block. Haplovalidate specifically estimates two key parameters of the haplotype reconstruction from the data, that is minimal cluster correlation and window size. Other haplotype reconstruction parameters were not inferred from the data, but chosen to fit to a broad range of data sets (see Table 1).

**TABLE 1:** haploReconstruct parameters and the values used for the ad hoc analysis and haplovalidate

Reconstruction parameter	Definition	Ad hoc	Haplovalidate
min.cl.cor	Minimum correlation threshold for SNP clustering	0.7	Set by haplovalidate
winsize	Window size	1 Mb	Set by haplovalidate (MNCS of 0.01 or 0.03)
min.minor.freq	Minimum starting allele frequency of SNPs used for clustering	0	0
max.minor.freq	Maximum starting allele frequency of SNPs used for clustering	0.03	1
minfreqchange	Minimum frequency change of SNPs used for clustering	0.2	0
minrepl	Minimum number of replicates in which SNP is present	2	1
min.cl.size	Minimum number of SNPs in a cluster	8	20
min.inter	Minimum number of SNPs that overlap for merging clusters	4	4

The ad hoc reconstruction parameters were taken from a standard configuration used by Franssen, Barton, et al. (2017).

### 2.3 Detailed procedure

**FIGURE 2:**
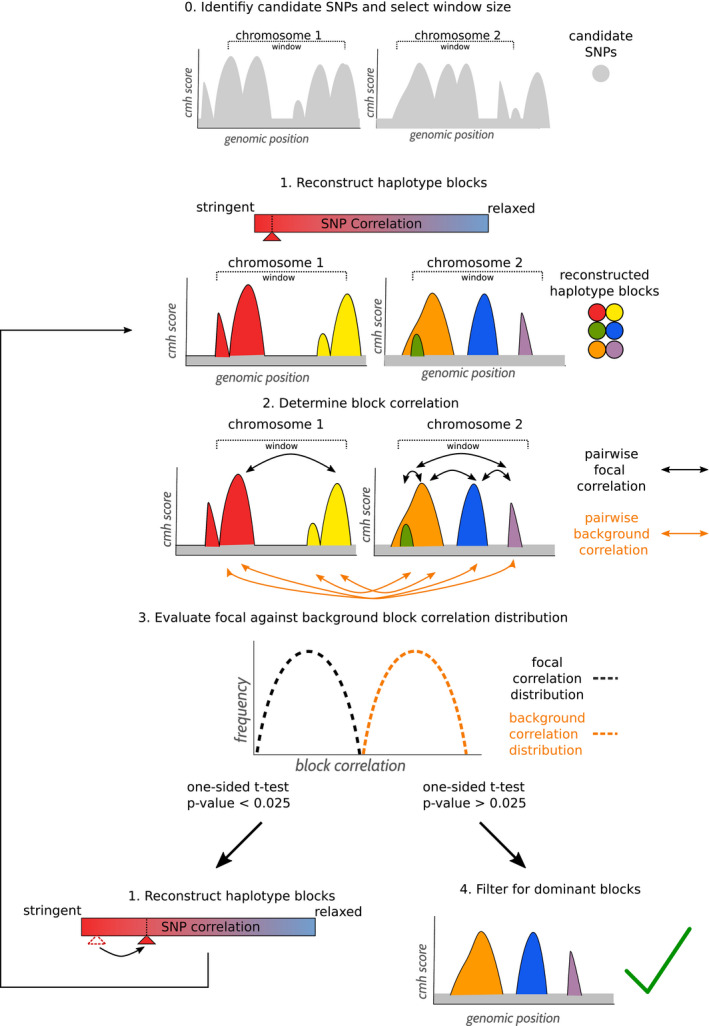
Overview of the iterative procedure to define haplotype blocks with haplovalidate. After identifying candidate SNPs and calculating the window size (step 0), the haplotype reconstruction starts with stringent parameters (step 1). The allele frequency trajectory correlation of SNPs from different blocks on the same chromosome (focal block correlation) is compared to the correlation of SNPs from blocks located on different chromosomes and tested for significant differences (step 2, 3). If focal block correlations are higher than the background correlations, this indicates that a too stringent correlation cut‐off was used; and haplotype reconstruction is repeated using less stringent parameter (back to step 1). If focal block correlations are similar to background correlations, the least significant haplotype reconstruction is used and regions with overlapping blocks are filtered for the most dominant block (step 4) [Colour figure can be viewed at wileyonlinelibrary.com]

Haplovalidate consists of five different steps (see Figure 2). Please find details on parameter choice and candidate SNP identification in the sections below.

#### 2.3.1 Step 0: Identify candidate SNPs and select window size

Candidate SNPs (i.e. SNPs that change more in frequency than expected under neutrality) were identified by chi‐square test and Cochran–Mantel–Haenszel (CMH) test comparing the most evolved to the founder population, and the optimal window size is calculated using the MNCS (median normalized CMH score sum) approach, which is explained in detail in the section *Variable parameters* below.

#### 2.3.2 Step 1: Reconstruct haplotype blocks

Haplotype blocks are reconstructed using parameters as described below and minimum cluster correlations ranging from 0.9 to 0.3 in 0.1 steps. The reconstruction with most reconstructed blocks is used as starting point to determine focal and background block correlations.

#### 2.3.3 Step 2: Determine focal and background block correlations

We normalize allele frequency data by using arcsine‐square‐root‐transformation followed by centring and scaling. Block correlations are then calculated for SNP allele frequency trajectories of any two haplotype block marker SNPs of two blocks (pairwise correlations), and the median is taken from all estimates of the two block comparison. The two blocks can be either blocks within the window used for haplotype reconstruction (focal block correlations) or blocks on different chromosomes (background block correlations). Focal block correlations can only be calculated if at least two blocks are present in a given window. Windows containing only one block are not considered. For blocks with more than 2000 SNPs, only 2000 randomly selected SNPs were used to increase computational efficiency.

#### 2.3.4 Step 3: Evaluate block correlation distributions

Block correlations are normalized using the Fisher transformation (Fisher, 1915). The difference in focal and background correlations is determined by a one‐sided *t* test. In the case of a significant difference between chromosome and background (*p*‐value <.025), the procedure is repeated with step 1 and a less stringent minimum cluster correlation (0.01 steps). If there is no significant difference between chromosome and background (*p*‐value ≥.025), haplovalidate uses the last significant haplotype reconstruction, therefore reducing nonindependent haplotype blocks to a minimum. If fewer than three values are available for focal or background block correlation, haplovalidate returns no result.

#### 2.3.5 Step 4: Filter for dominant blocks

If a selection target is present in several haplotype blocks, they will be identified as independent, overlapping blocks. To identify the dominating block per selection target, we filter genomic regions with overlapping blocks for the block with the most significant allele frequency change (CMH test/chi‐square test; Spitzer, Pelizzola, & Futschik, 2020).

### 2.4 Variable parameters

The parameter ‘minimal cluster correlation’ determines the cut‐off for hierarchical clustering and is among the most important parameters for haplotype reconstruction. Using too high correlation coefficients splits a selected haplotype block into smaller regions, giving the impression of multiple selection targets, rather than one. If the correlation coefficients are too low, independent selected haplotype blocks are combined and consequently the number of selection targets is underestimated (Franssen, Barton, et al., 2017). However, the definition of too high and too low varies between different data sets as the correlation between marker SNPs of a selected haplotype block depends on the strength of selection and the initial frequency of marker SNPs. Haplovalidate infers the optimal haplotype block reconstruction using data‐specific minimal cluster correlations. This is achieved by using a cluster correlation, which is just above random correlations. More specifically, we calculate the median correlation between two marker SNPs from two haplotype blocks on the same chromosome (focal block correlation) and two haplotype block marker SNPs between chromosomes (background block correlation) for each minimal cluster correlation tested. Because different chromosomes are not physically linked, background block correlations are on average an estimate of random associations and serve as a null distribution. Focal block correlations higher than background correlation (*p*‐value <.025) suggest that the two blocks should be joined. In contrast, the focal block correlation of reconstructed haplotype blocks belonging to independent selection targets will not differ from the null distribution. We caution that LD for short blocks tends to be higher than background LD between chromosomes. This may cause a more liberal combination of haplotype blocks in very close distance. Since reconstructed haplotype blocks of simulated and experimental data are typically considerably larger than the regions of elevated LD (see Figures S1 and S2), we do not consider this a major problem of our approach.

The parameter window size determines the upper length of reconstructed haplotype blocks. Small windows result in shorter blocks (Franssen, Barton, et al., 2017), which facilitate the separation of independently selected regions. However, reliable haplotype block reconstruction strongly relies on the number and effect size of the candidate SNPs. As the number and effect size of candidate SNPs are highly data‐specific, we do not only consider the number and effect size of SNPs in a given window, but also take the total number of candidate SNPs into account. Hence, we use the same fraction of total candidate SNP effect size per window and therefore make haplotype reconstruction comparable, even for data sets that differ in candidate SNP number and/or effect size distribution. Consequently, a few moderately significant candidate SNPs may be sufficient to define a haplotype block when no other candidate SNPs are present on the chromosome. Alternatively, with many candidate SNPs, larger windows with more and highly significant candidate SNPs are needed to reconstruct a haplotype block. This is achieved with the *median normalized CMH score sum* (MNCS), a normalized measure combining effect sizes (based on the CMH test) and SNP numbers associated with window size (see also Equation (1) where *p* is the *p*‐value of the CMH test and −log(*p*) the CMH score):

We split the candidate SNPs in windows of a given size (0.1–10 Mb in 0.1 Mb steps). For each window, we summed the CMH scores of the candidate SNPs and divided it by the sum of all CMH scores. From the distribution of all normalized window sums, we took the median, that is MNCS.

(1) $$\text{MNCS}=\text{median}(\left(\sum ‐\log{\left(p\right)}_{\text{window}})/\left(\sum ‐\log{\left(p\right)}_{\text{total}}\right)\right)$$

Standard haplovalidate analyses use a window size corresponding to a MNCS of 1%, as this fraction has proven to give good results for most data sets. In the case haplovalidate fails because there are not enough SNPs in a window we repeated the analysis with a MNCS of 3% and the corresponding window size.

### 2.5 Fixed parameters

We fixed the haplotype reconstruction parameters such that alleles starting from any frequency (starting allele frequency between 0 and 1) in at least one replicate are included. The allele frequency change threshold parameter aims to focus on SNPs changing more than expected under drift. As we used a modified chi‐square test and CMH test that have a null hypothesis adapted for drift and pool sequencing noise (Spitzer et al., 2020), we set the allele frequency change threshold to 0. Following Barghi et al. (2019), we required at least 20 SNPs for each cluster and only clusters sharing at least four SNPs could be merged.

### 2.6 Candidate SNPs

Because only selected haplotype blocks are of interest, clustering is performed only on candidate SNPs which change more in frequency than expected under neutrality. Candidate SNPs were identified by chi‐square test and Cochran–Mantel–Haenszel (CMH) test accounting for drift and pool sequencing variance as implemented in the r package ACER (Spitzer et al., 2020). All available time points were used for calculating the test statistics. Effective population size was calculated (estimatewndne function from poolseq package; Taus et al., 2017) for intermediate and most evolved generations. Here, allele frequency shifts between the starting and the focal generation were used to estimate standardized variance (Jónás, Taus, Kosiol, Schlötterer, & Futschik, 2016). In addition, we corrected the *p*‐values for multiple testing using the Benjamini–Hochberg method as implemented in the r function p.adjust. We chose candidate SNPs with a corrected *p*‐value < .05 in the CMH test or *p*‐value < .001 in the chi‐square test of any replicate. Using both tests allows for including replicate‐specific responses, which could be missed by the CMH test alone as it detects consistent changes across replicates. A more stringent *p*‐value threshold for the chi‐square test was used to include only replicate‐specific candidates with a pronounced allele frequency change. If a simulation run had more than 50,000 candidate SNPs per chromosome, we randomly sampled 50,000 SNPs from the candidates to increase computational efficiency.

### 2.7 Identification of multiple‐target haplotype blocks

We identified multiple‐target haplotype blocks by comparing haplotype blocks reconstructed based on all generations (up to 60) to those obtained from reconstructions using time points up to generation 20 (or generation 30 if not enough candidate SNPs were identified at F20). The presence of regions with several haplotype blocks at an intermediate time point but only a single haplotype block in the later generations is considered the signature of a multiple‐target haplotype (Figure 8). We account for this by replacing the single haplotype block from the later time points with the haplotype blocks identified at the intermediate time points.

The results of haplovalidate with and without intermediate generations were compared based on each of the normalized summary statistics (i.e. true‐positive rate, single target fraction, multiple‐target fraction, multiple target per haplotype fraction, false‐positive rate, Figure 9) using arcsine‐square‐root‐transformation and Welch's *t* test.

### 2.8 Simulations

We performed 1,000 genome‐scale forward simulations covering the two main autosomes of *Drosophila simulans* using mimicree2 version 206 (Vlachos & Kofler, 2018). We simulated a diploid sexual organism and loci with constant selection coefficients (s) and a dominance coefficient (h) of 0.5. Fitness (w) of genotypes was defined as follows: waa = 1, waA = 1 + sh, wAA = 1 + s. We simulated selective sweeps, rather than a quantitative trait experiencing a shift in trait optimum, as most E&R studies only focus on the early phase of adaptation where the trajectories of QTLs and selective sweeps are very similar (Barghi & Schlötterer, 2020). We mimicked an Evolve and Resequence (E&R) experiment in *D. simulans* with 10 replicates, each with a population size of 1,200, evolving for 60 generations. We extracted allele frequencies for every 10th generation (sync file format; Kofler, Pandey, & Schlötterer, 2011) and haplotypes for the most evolved generation (F60). We restricted our analysis to the main autosomes of *D. simulans* (chromosome 2 and chromosome 3). The founder population was created from 189 experimentally phased haplotypes originating from a natural *D. simulans* population (Barghi et al., 2019). We generated the same number of simulations for 16, 32, 64 or 128 selected loci (equal number of loci on both chromosomes). Starting allele frequencies and selection coefficients were taken from 99 selection targets detected in a *Drosophila* E&R study (Barghi et al., 2019). In the case of 128 loci, 29 randomly chosen estimates for selection coefficients and starting allele frequency were used twice. SNPs matching the allele frequency were randomly chosen from the founder population. Selection coefficients ranged from 0.02 to 0.14, which covers the lower boundary of detectable effect sizes (Baldwin‐Brown, Long, & Thornton, 2014; Kofler & Schlötterer, 2014). Starting allele frequencies ranged from 0.003 to 0.76 with low‐frequency alleles pairing with both, high and low selection coefficients, indicating that small and big effect alleles were used for our simulations. The corresponding number of loci was randomly drawn from the set of 99 selection targets without replacement. We used the *D. simulans*‐specific recombination map (Dsim_recombination_map_LOESS_100kb_1.txt; Howie, Mazzucco, Taus, Nolte, & Schlötterer, 2019). We generated ‘Pool‐seq data’ with 50× coverage and added sequencing noise by binomial sampling based on the allele frequencies.

### 2.9 Bottleneck simulations

As a decrease in population size can severely influence haplotype structure in a population, we tested the performance of haplovalidate on E&R simulations with two bottleneck scenarios. We repeated 100 simulations from the scenario with 32 targets and used the same simulation parameters but reduced the population size from generation 20 to 30 to 20% or 10%.

### 2.10 Application to experimental data

We applied haplovalidate to two different *Drosophila* E&R data sets which capture experimental evolution to a new temperature regime in *D. simulans* but show different selection responses. Barghi et al. (2019) found 88 selected regions on the autosomes, whereas Mallard, Nolte, Tobler, Kapun, and Schlötterer (2018) focused on two regions while analysing the top 100 CMH test outlier SNPs. For an unbiased comparison of the clustering results, we aimed to use the same candidate SNPs as in the original studies. As the top 100 CMH test outlier SNPs did not result in enough SNPs per window to perform haplotype reconstruction for the data of Mallard et al. (2018), we extended the SNP‐set to the top 1,000 outlier SNPs per chromosome.

We performed a permutation test to evaluate whether the number of shared haplotype blocks between the data of Barghi et al. (2019) or Mallard et al. (2018), and our method exceeds random expectations. To this end, we randomly sampled from the candidate SNPs of each data set the same number of SNPs as present in the haplotype blocks. We then computed the maximum number of haplotype blocks sharing at least 50% of the candidate SNPs between the resampled Portugal and Florida data (*N* = 5,000). Permuted and observed data were compared using a chi‐square test.

## 3 RESULTS

### 3.1 Performance

An approach for reconstructing selected haplotype blocks without information about the founder haplotypes was proposed by Franssen, Barton, et al. (2017) and implemented in the r package haploReconstruct. This approach uses window‐based correlation analysis of allele frequency data across replicates and time points combined with hierarchical clustering. Each cluster of SNPs corresponds to a selected haplotype block. Franssen, Barton, et al. (2017) successfully identified selected haplotype blocks from simulated and experimental E&R data. The reconstructed haplotype blocks were validated using haplotype sequence data. Most importantly, the approach of Franssen, Barton, et al. (2017) requires a low starting frequency of the selected haplotype block. While this is typically the case, also higher starting frequencies need to be considered (Barghi et al., 2019). To test the generality of the approach and parameters proposed by Franssen, Barton, et al. (2017), we applied them to E&R data simulated with a broader range of parameters. We used a set of 189 sequenced *D. simulans* haplotypes (chromosome 2 and 3) to create a founder population (*N* = 1,000) with realistic linkage disequilibrium. Selection coefficients and starting allele frequencies were matched to a *Drosophila* E&R experiment by Barghi et al. (2019). We tested four different numbers of selected loci (16, 32, 64 or 128 loci per chromosome), randomly sampling SNPs with the frequency in the founder population matching Barghi et al. (2019). Simulations were conducted over 60 generations in 10 replicates using a selective sweep scenario.

**FIGURE 3:**
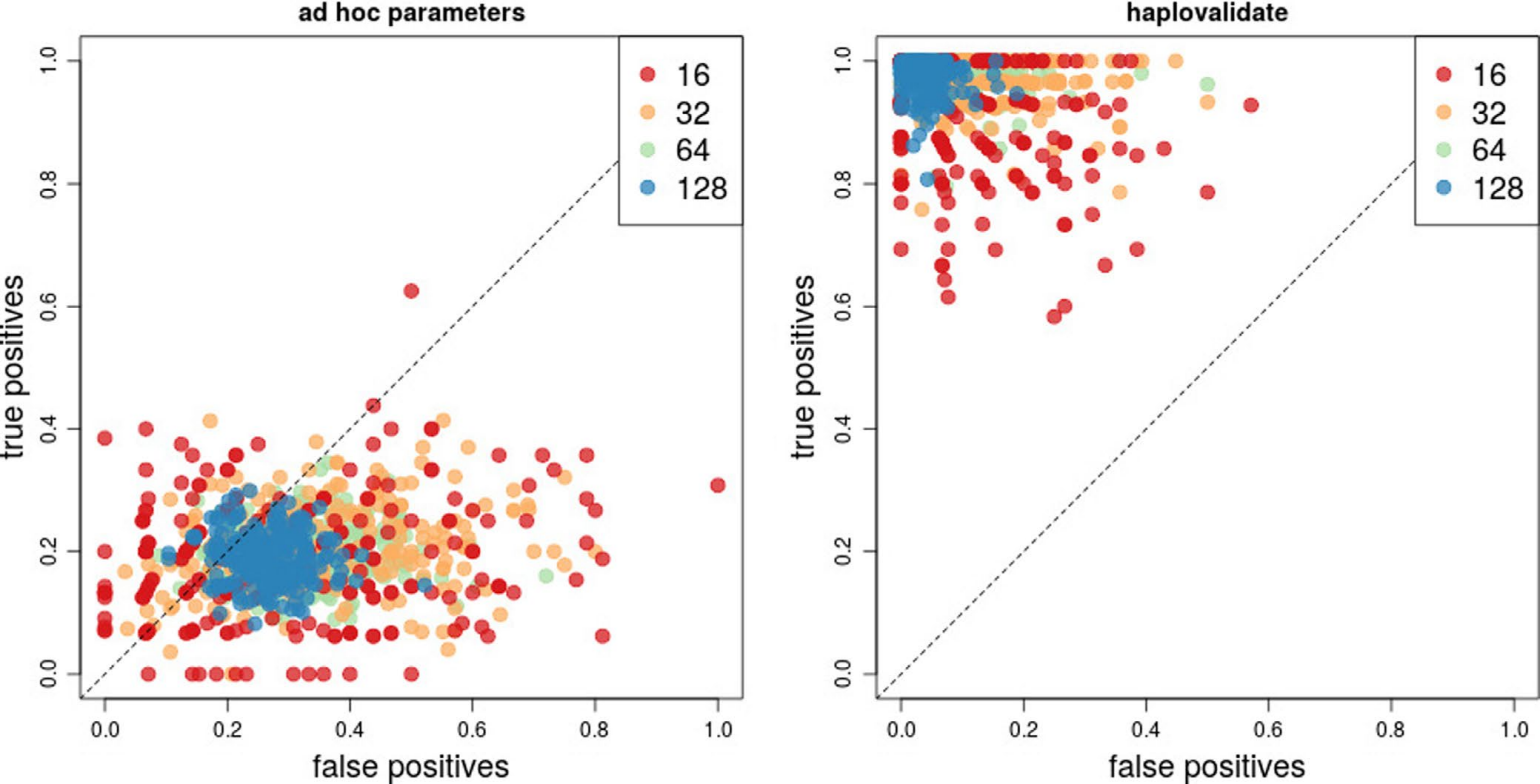
True‐ and false‐positive rates of reconstructed selected haplotypes based on the parameters proposed by Franssen, Barton, et al. (2017) for low starting frequencies (left panel) and on haplovalidate (right panel) using 1,000 selective sweep simulations with a broad range of starting frequencies for a different number of selected loci (see colour code in legend) [Colour figure can be viewed at wileyonlinelibrary.com]

Using the reconstruction parameters for selection targets with low starting frequencies as recommended by Franssen, Barton, et al. (2017), 19.8% (median true‐positive rate over all simulations) of the selected loci were located in a reconstructed haplotype block. The false‐positive rate, that is the detection of blocks without a selected locus, exceeded the true‐positive rate (29.6% median false‐positive rate over all simulations, Figure 3, left panel). Apparently, these parameters were not suitable for the reconstruction of selected haplotype blocks from simulations with multiple selected loci with variable starting frequencies, illustrating that haplotype reconstruction parameters are highly dependent on the analysed data set. Using the same set of simulations for validation as described above, haplovalidate is able to recover selected haplotype blocks covering 97% of the selected loci (median true‐positive rate over all simulations) with only 7% of the reconstructed blocks not containing a selected locus (median false‐positive rate over all simulations, Figure 3, right panel).

**FIGURE 4:**
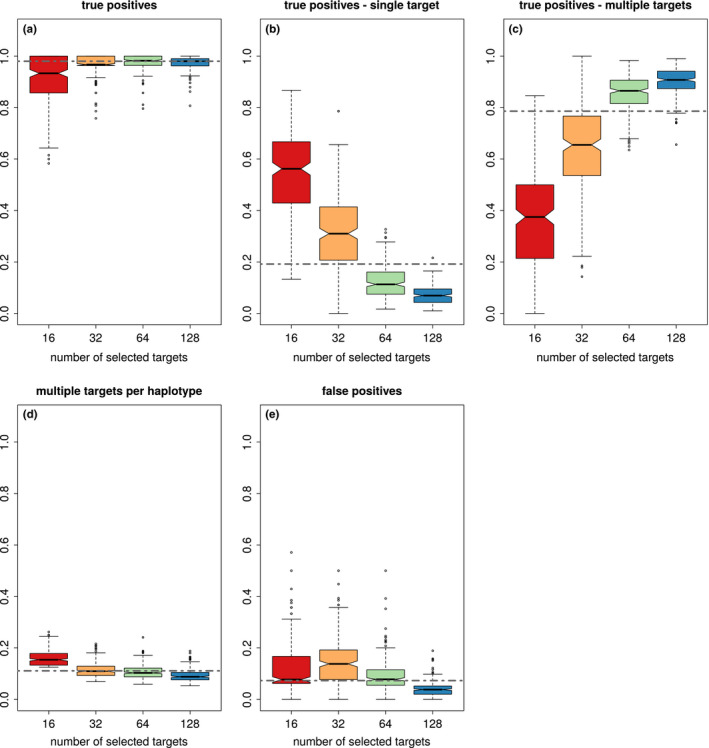
The performance of haplovalidate based on 1,000 selective sweep simulations containing either 16, 32, 64 or 128 selected alleles. (a) True‐positive rate (all identified selection targets), (b) fraction of identified single targets of selection, (c) fraction of selected targets sharing a haplotype block, (d) average fraction of selection targets per haplotype block, (e) false‐positive rate. Dashed line represents the marginal median across different number of selection targets [Colour figure can be viewed at wileyonlinelibrary.com]

**FIGURE 5:**
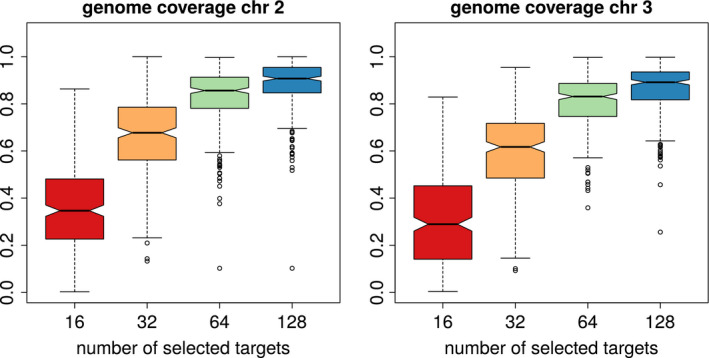
Depending on the number of selection targets, selected haplotype blocks span a moderate to high fraction of the genome as show for chromosome 2 (left) and 3 (right) [Colour figure can be viewed at wileyonlinelibrary.com]

Analysing the haplovalidate performance in more detail shows that haplovalidate indeed reliably detects haplotype blocks for simulations with different numbers of selection targets; 93%–98% of all selected alleles were captured (Figure 4a) with a low false‐positive rate, ranging from 4% to 14% (Figure 4e). Selected haplotype blocks covered moderate to high proportions of the chromosomes depending on the number of selection targets and their starting frequencies. While one third of the chromosomes was covered in the case of 16 selection targets, up to 90% of the genome could be covered by selected haplotype blocks when 128 targets were present (Figure 5). Not every selected allele resulted in an independent haplotype block—only 7%–56% of the selected alleles did so (Figure 4b) because many inferred haplotype blocks contain more than one target of selection. The fraction of selected alleles on haplotype blocks with multiple targets can be substantial (median 38% to −91%, see Figure 4c) with a given haplotype block containing a median of 7%–14% of the targets (see Figure 4d). The issue of multiple selection targets being present on a single haplotype block becomes increasingly important with the number of selected loci in a simulation.

**FIGURE 6:**
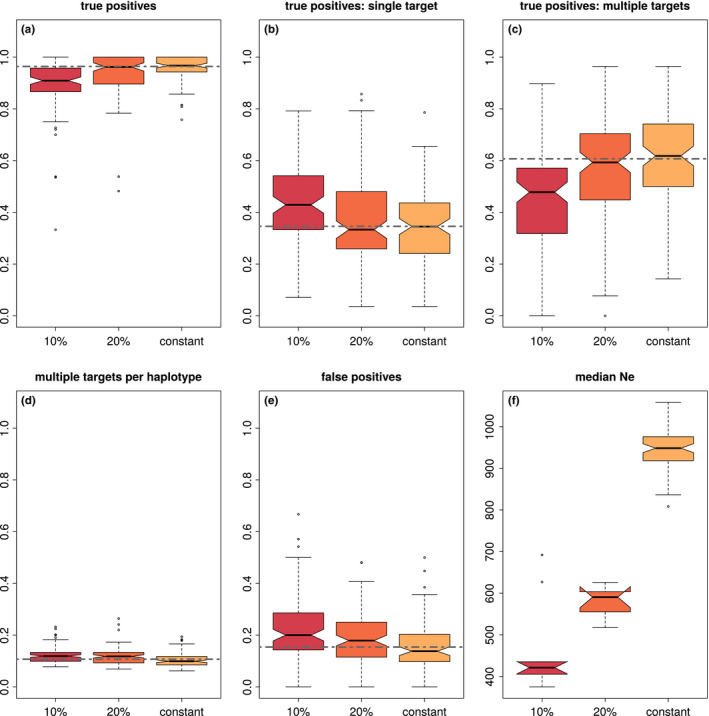
The performance of haplovalidate is robust to fluctuations in population size. 100 sweep simulations with 32 targets were performed with constant population size or containing a bottleneck which reduces the initial population size to 20% or 10% for 10 generations. (a) True‐positive rate (all identified selection targets), (b) fraction of identified single targets of selection, (c) fraction of selected targets sharing a haplotype block, (d) average fraction of selection targets per haplotype block, (e) false‐positive rate, (f) median effective population size. Dashed line represents the marginal median across simulations with different population sizes [Colour figure can be viewed at wileyonlinelibrary.com]

In addition, we compared the performance of haplovalidate in simulations with and without bottleneck. We analysed two different bottleneck scenarios, reducing the population size to 20% or 10% for ten generations. Although the resulting differences in effective population size were rather large (Figure 6 panel h), the total true‐positive identification rate was still above 90% for both bottleneck scenarios. Overall, we identified slightly less selection targets (Wilcoxon rank‐sum test *p*‐value < .001, Figure 6 panel a), which reflects a weaker selection signature due to the loss of selected haplotype blocks in the bottleneck (Figure 6 panel h). This also resulted in a slightly higher false‐positive rate (Figure 6 panel e, Wilcoxon rank‐sum test *p*‐value < .001 for 10% and *p*‐value < .05 for 20%). Interestingly, the stronger bottleneck scenario resulted in an increase of single target and a decrease of multiple‐target identifications (Figure 6 panel b,c), indicating that haplotype blocks with multiple selection targets are more frequently lost due to their lower initial frequency in the population (see below). We conclude that haplovalidate is robust to changes in population size, reliably detecting selected haplotype blocks even after severe population reductions.

### 3.2 Using early generations to identify blocks with multiple selection targets

**FIGURE 7:**
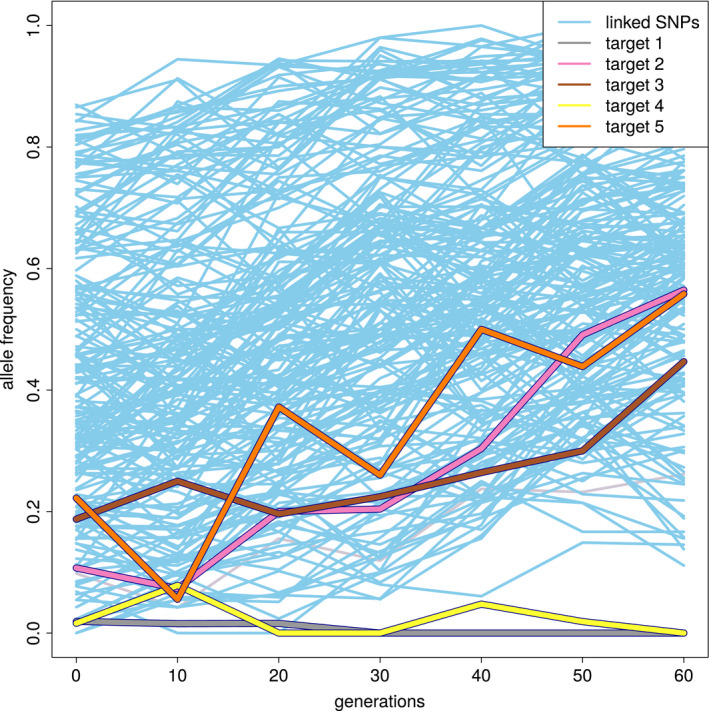
Allele frequency trajectories of a reconstructed haplotype block in one replicate over 60 generations with selection targets marked in different colours (simulated data). Candidate SNPs linked to the selection targets are marked in blue [Colour figure can be viewed at wileyonlinelibrary.com]

**FIGURE 8:**
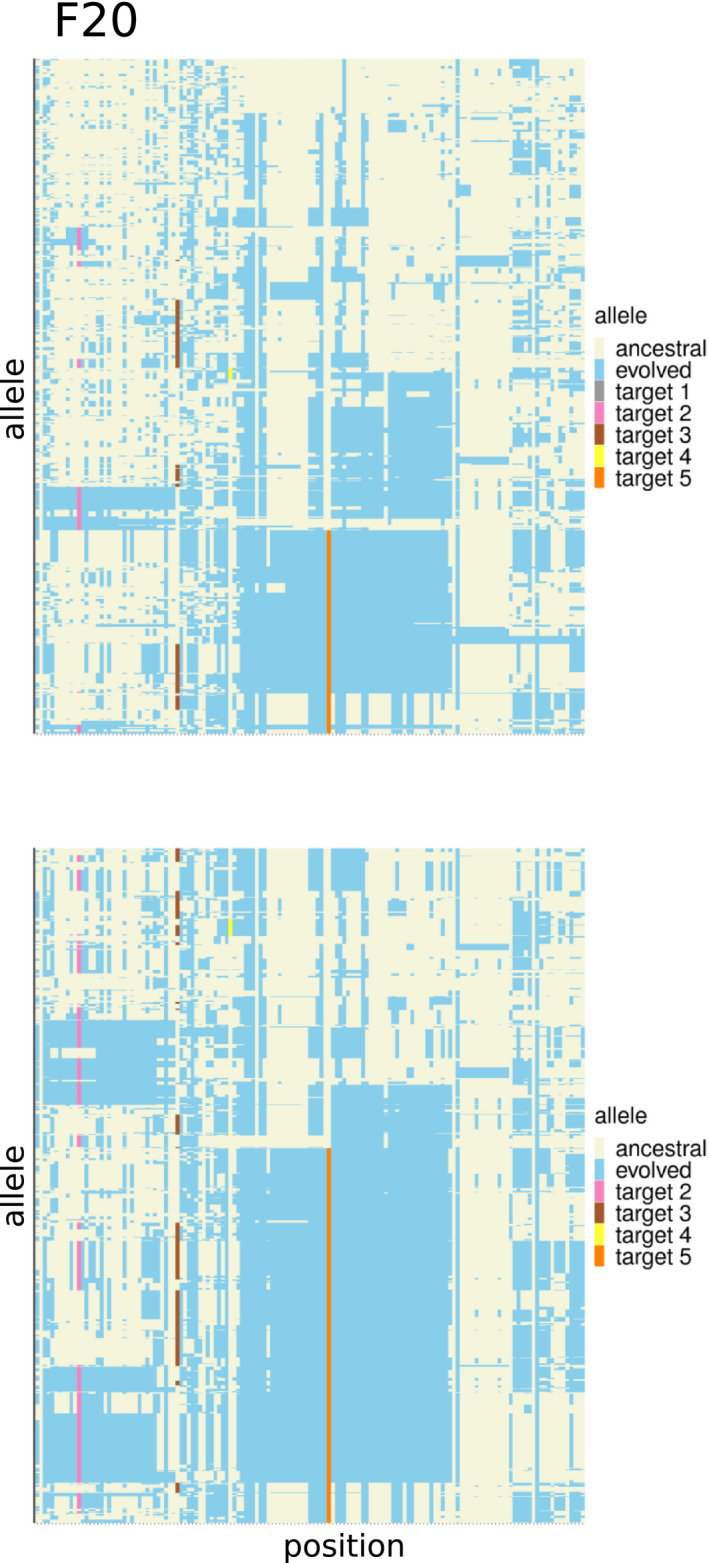
Haplotype blocks containing multiple selected alleles behave as single target of selection (see Figure 7 for the corresponding allele frequency trajectories, simulated data). Each row indicates a haplotype each column indicates a genomic position with a polymorphic site. Haplotypes present in F20 (top) and F60 in one replicate of a region containing five targets of selection. Alleles present in the evolved reconstructed haplotype block are marked in blue (other colours for the selected alleles); ancestral alleles are light yellow [Colour figure can be viewed at wileyonlinelibrary.com]

The allele frequency trajectory of multiple selected SNPs in high linkage disequilibrium (LD) cannot be easily distinguished from a single selection target. Because of the additive effect of the selection targets, highly correlated allele frequency trajectories will be obtained (see Figure 7) even if the selected SNPs are not in high LD at the beginning of the experiment: The haplotype block with the largest number of selection targets will increase most strongly and LD will increase during the experiment (see Figure 8). Even if the selected alleles have a low starting frequency and are not linked, recombination during the experiment may generate haplotype blocks with multiple selection targets, which will experience a stronger selective advantage, resulting again in increased LD. Thus, haplovalidate is very likely to reconstruct haplotype blocks with multiple targets, in particular for simulations with many selected alleles and high starting frequencies.

Across all simulations 37,846 selected alleles (82%) were located on a haplotype with multiple selected alleles at generation 60. 95% of these alleles share haplotypes already in the founder population. This is significantly more often than random pairs of 35,000 SNPs having the same physical distance (chi‐square test, *p*‐value < .001). This result indicates that haplotype structure in the founder population predetermines the occurrence of multiple‐target haplotype blocks.

Given that LD between selection targets increases during the experiment, we reasoned that haplotype block reconstruction at earlier generations may be more powerful to distinguish independent selection targets that have high LD at later stages of the experiments. As expected, we were able to fine‐map multiple‐target blocks by comparing reconstructions based on all generations (up to 60) to those obtained from intermediate time points up to generation 20 (or generation 30 if not enough candidate SNPs were identified at F20). As in the majority of cases the multiple‐target haplotype is rare compared to the single target haplotypes in the founder population, single target haplotypes have more pronounced allele frequency differences in the intermediate generations. However, the additive selective advantage of all selected alleles on a haplotype block ultimately outcompetes single‐target haplotypes if not lost by genetic drift. The characteristic hallmark of reconstructed haplotype blocks with multiple selection targets is the presence of a single haplotype block at generation 60, but several reconstructed haplotype blocks at intermediate time points.

**FIGURE 9:**
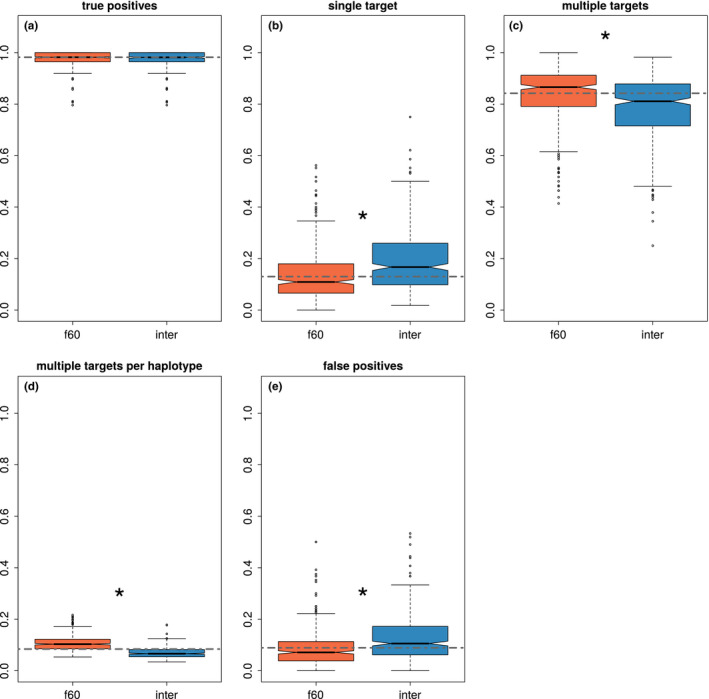
Haplovalidate with and without including intermediate time points. (a) True‐positive rate (all identified selection targets), (b) fraction of haplotype blocks with a single selection target, (c) fraction of haplotype blocks with multiple targets, (d) average fraction of selection targets on a haplotype block, (e) false‐positive rate. Dashed line represents the overall median for each parameter. Asterisks indicate significant differences for clustering with and without intermediate time points (*p*‐value < .05) [Colour figure can be viewed at wileyonlinelibrary.com]

Haplotype reconstruction of intermediate time points can only be informative when a sufficient number of candidate SNPs is available for clustering. Seventy‐four simulations with 32 targets, 166 with 64 targets and 126 with 128 targets contained sufficient candidate SNPs for the analysis of intermediate time points. The inclusion of intermediate time points increased the number of haplotype blocks while decreasing the average number of selection targets per haplotype block (see Figure 9). This was also observed when simulations with different numbers of selection targets were analysed separately (see Appendix S3).

### 3.3 Experimental data

We applied haplovalidate to two different *D. simulans* E&R data sets which differ in their selection response to a new temperature regime. Barghi et al. (2019) found 88 selected regions on the autosomes whereas Mallard et al. (2018) highlighted five selection targets.

**FIGURE 10:**
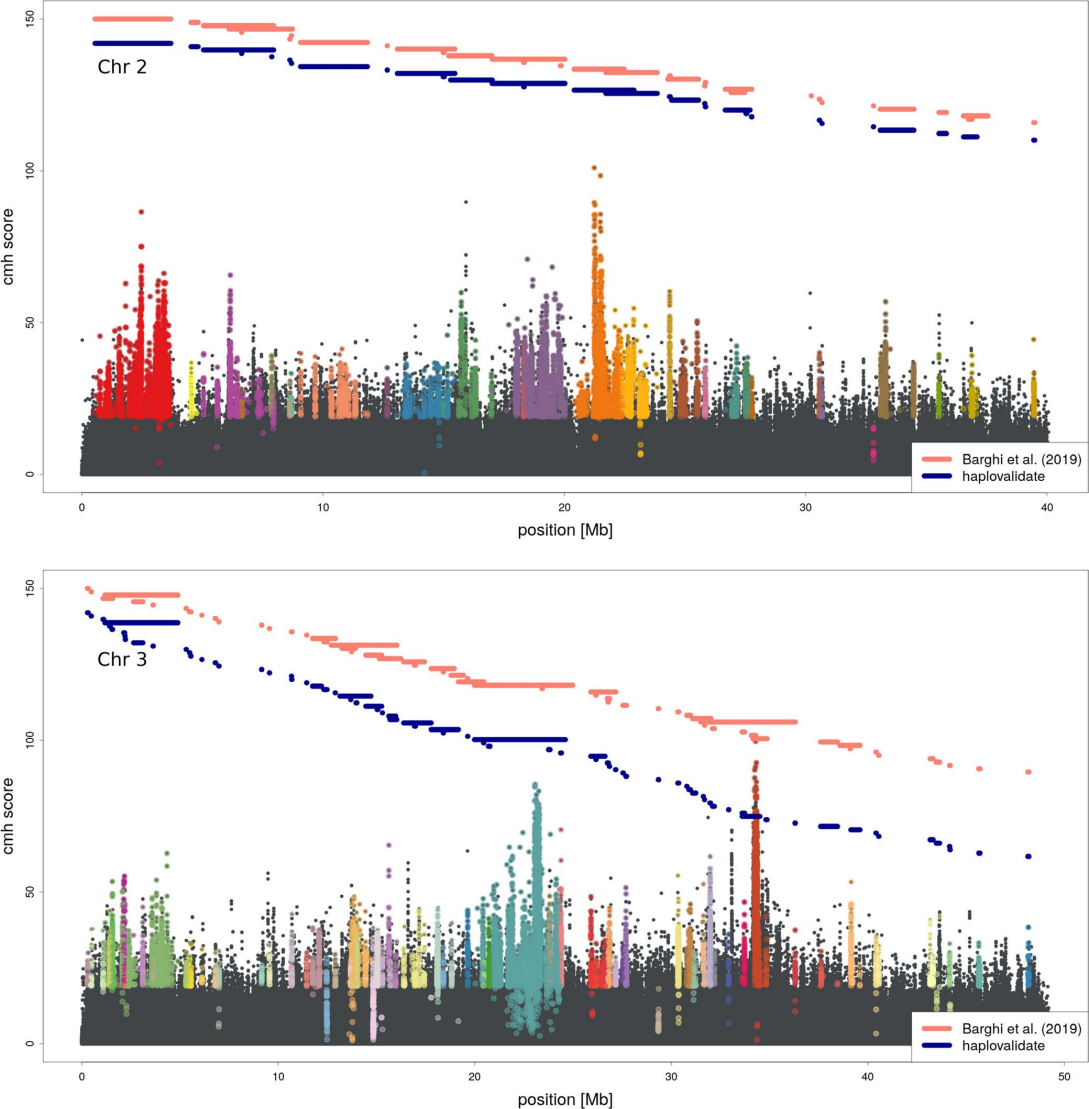
Manhattan plot for the data of Barghi et al. (2019). SNPs belonging to haplotype blocks identified by haplovalidate are shown in different colours. The horizontal lines show the genomic regions spanned by haplotype blocks of Barghi et al. (2019) (pink) and haplovalidate (blue) [Colour figure can be viewed at wileyonlinelibrary.com]

Because the haplotypes reconstructed by Barghi et al. (2019) were validated with experimentally derived haplotypes from founder and evolved generations, we consider these results as a gold standard, against which we test the performance of haplovalidate. Using the candidate SNPs from Barghi et al. (2019) with haplovalidate identified similar haplotype blocks. Instead of 88 blocks, haplovalidate detected 104 haplotype blocks of which all 104 overlap at least for 50% with regions from Barghi et al. (2019). Vice versa, 70 regions detected by Barghi et al. (2019) overlap at least for 50% with haplovalidate (see Figure 10). In both cases, the overlap is significantly higher than expected by chance (chi‐square test *p*‐value < .001). Interestingly, on chromosome 2 the reconstruction of haplovalidate is almost indistinguishable from Barghi et al. (2019). On chromosome 3, more independent haplotype blocks are inferred by haplovalidate. Based on the currently available data, it is not possible to distinguish whether haplovalidate is more powerful to detect independent selection targets, or whether some of the additional haplotype blocks are false positives not containing a selection target.

**FIGURE 11:**
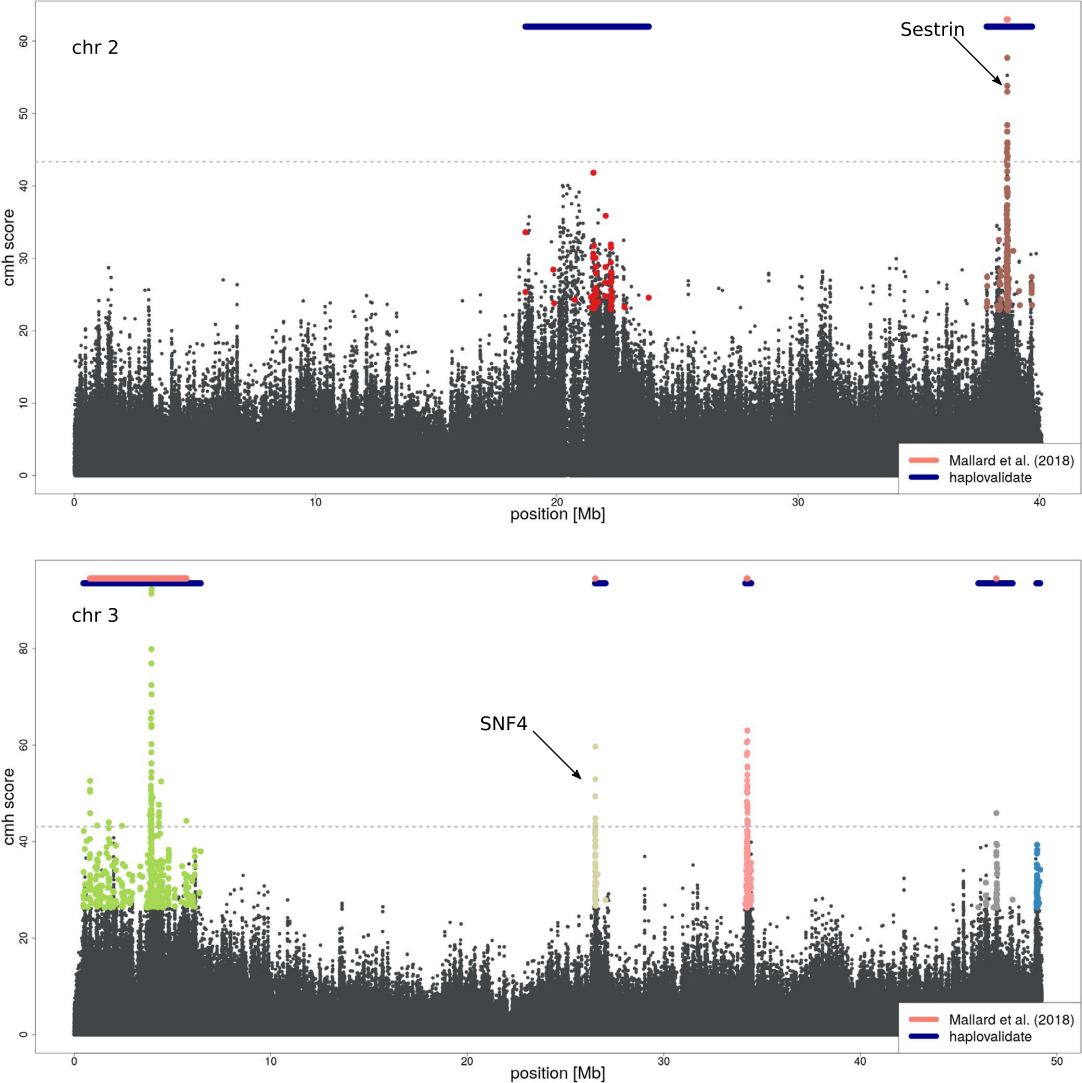
Manhattan plot for the data of Mallard et al. (2018) on chromosome 2 (top) and chromosome 3 (bottom). The horizontal lines show the genomic regions spanned by haplotype blocks of Mallard et al. (2018) (pink) and haplovalidate (blue). The dashed line represents the significance threshold used by Mallard et al. (2018). The two top candidate genes (Sestrin and SNF4) identified by Mallard et al. (2018) are also highlighted [Colour figure can be viewed at wileyonlinelibrary.com]

We also applied haplovalidate to the data of Mallard et al. (2018). Based on the 1,000 most significant SNPs for each chromosome, haplovalidate identified all five regions detected in the original study (Figure 11), which is significantly more than expected by chance (chi‐square test *p*‐value = .03). In addition, we also identified two additional haplotype blocks, which are, however, not containing candidate SNPs reaching the significance threshold applied by Mallard et al. (2018).

## 4 DISCUSSION & OUTLOOK

Moving from a SNP‐centric analysis to the identification of selected haplotype blocks provides a significant advancement of E&R studies (Barghi & Schlötterer, 2019). Introducing haplovalidate, we provide a tool to make the reconstruction of selected haplotype blocks a routine method that does not rely on the availability of haplotype information from the founder population or from evolved individuals. We demonstrated with simulated and experimental data that haplovalidate can be applied to a broad range of data, from few to many targets of selection and with fluctuations in population size. We attribute this to the data‐driven selection of two key parameters of the reconstruction procedure, the minimum correlation between SNPs constituting a cluster and the window size. It is important to note that we only simulated selective sweeps and not a quantitative trait experiencing a shift in trait optimum. Since the allele frequency trajectories of sweeps and quantitative traits diverge after a trait optimum has been reached (Franssen, Kofler, & Schlötterer, 2017), the performance of haplovalidate is not clear when very advanced generations are included. Nevertheless, since the trajectories of sweeps and quantitative traits are rather similar until the new trait optimum is being reached (Franssen, Kofler, et al., 2017) and most E&R experiments only involve a moderate number of generations, we restricted our analysis to selective sweeps. Because *Drosophila* is the most commonly used out‐crossing model in E&R studies, we matched our simulation parameters to *D. simulans*, which is better suited for E&R studies than *D. melanogaster* (Barghi, Tobler, Nolte, & Schlötterer, 2017). We anticipate that haplovalidate can be also applied to E&R studies using other organisms with a different recombination landscape, such as *Caenorhabditis remanei* (Teotónio, Estes, Phillips, & Baer, 2017), because haplovalidate accounts for linkage by comparing the correlation within blocks and between blocks.

Our study also demonstrated the limits of a haplotype block‐based analysis of the adaptive architecture. We found that a high fraction of the reconstructed haplotype blocks contained multiple selected alleles. Interestingly, a similar observation was made by Sachdeva and Barton (2018) when analysing linked polygenic selection. In concordance with our study, the authors found that multiple‐target haplotypes outcompeted other haplotypes over time. The occurrence of multiple‐target haplotypes can lead to an underestimation of selection targets if each haplotype block is considered the outcome of selection operating on a single target in this block. However, we also show that restricting the analysis to intermediate generations (up to F20 or F30) improves the resolution—several multiple‐target blocks could be broken into single‐target blocks by the inclusion of earlier time points. Interestingly, most multiple‐target haplotype blocks were already present in the founder population indicating that the initial haplotype structure is an important factor for shaping the genomic signatures of adaptation, a result also supported by a theoretical study of Weissman and Barton (2012). More work is needed to understand how the haplotype composition of the founder population in combination with the number of founder haplotypes affects the power of E&R studies.

## AUTHOR CONTRIBUTIONS

K.A.O. and C.S. conceived the study and wrote the manuscript. K.A.O. performed the simulations and analysed the data.

## Supporting information

Supplementary Material

## Data Availability

The r package haplovalidate is available at https://github.com/popgenvienna/haplovalidate ([dataset] Otte and Schlötterer, 2020b). Haplotype block results, summary statistics and scripts for performing simulations and data analysis are available from the Dryad Digital Repository under https://doi.org/10.5061/dryad.3tx95x6c0 ([dataset] Otte and Schlötterer, 2020a).
