# Supplementary material for: Detecting selected haplotype blocks in evolve and resequence experiments
Source: Mol Ecol Resour. 2020 Sep 6;21(1):93–109. doi: 10.1111/1755-0998.13244 (PMC7754423; doi:10.1111/1755-0998.13244)
Supplement: Supplementary file 1 — Supplementary Material [file MEN-21-93-s001.pdf]

Detecting selected haplotype blocks in Evolve and Resequence experiments

Kathrin A. Otte, Christian Schlötterer

Institut für Populationsgenetik, Vetmeduni Vienna, Vienna, Austria

Supplementary Data

S1 – Median block size of reconstructed haplotypes

Benchmark Simulations F60

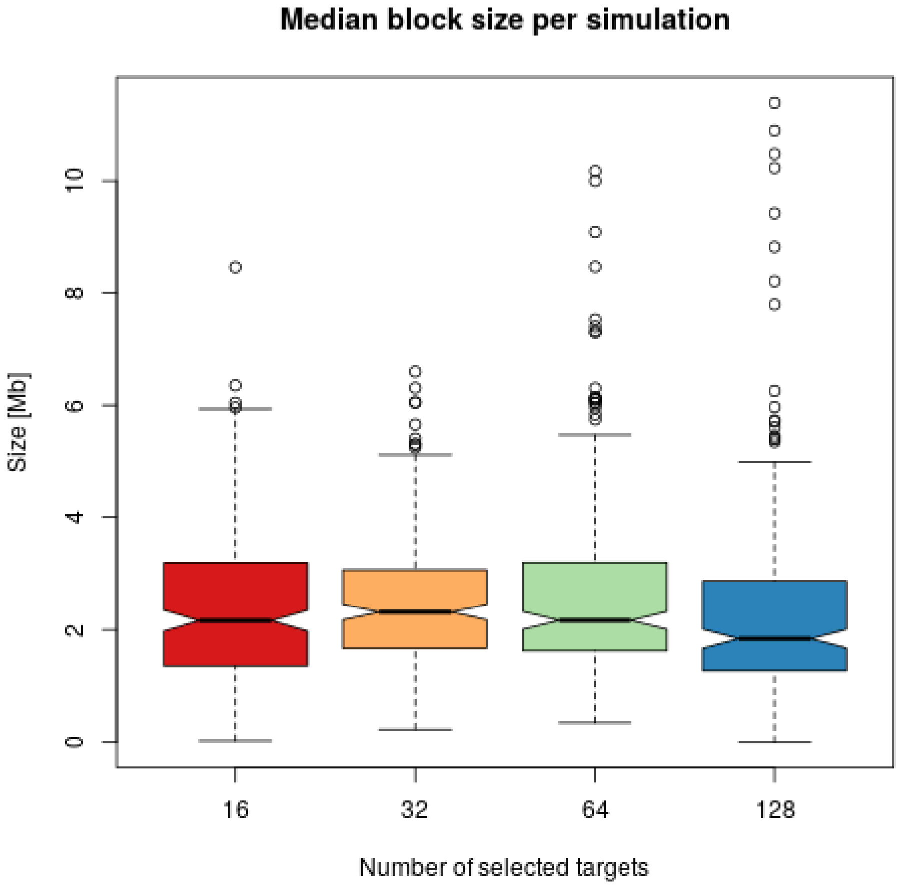

Figure 1: Median haplotype block size per simulation for simulations with 16, 32, 64 or 128 selection targets.

Experimental Data

Barghi et al. (2019) median bock size : 2.5 Mb

Mallard et al. (2018) median bock size : 5.1 Mb

S2 – Linkage Disequilibrium

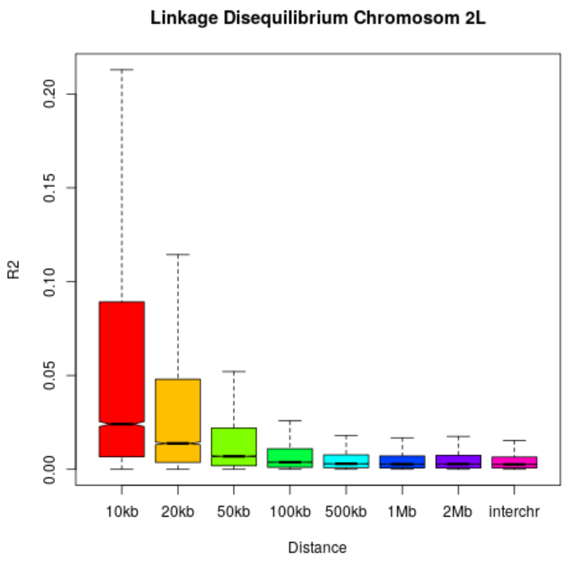

Figure 2: Linkage disequilibrium of chromosome 2L in 189 individual D. simulans haplotypes (Barghi et al., 2019) as measured by r2 for different distance bins (0-10 kb, 10-20kb etc.) and interchromosomal (interchr) r2. Each bin contains 1000 randomly chosen r2 estimates. Outliers are not shown.

S3 – Intermediate generation analysis for different numbers of selection targets

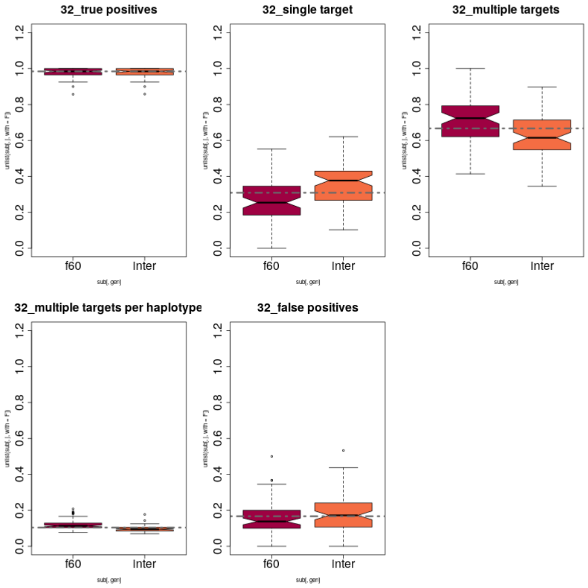

Figure 3: Haplovalidate with and without including intermediate time-points for simulations containing 32 selection targets. A: True positive rate, B: fraction of selected regions with a single selection target, C: fraction of haplotype blocks with multiple targets, D average number of selection targets on a haplotype block, E: false positive rate. Dashed line represents the overall median for each parameter.

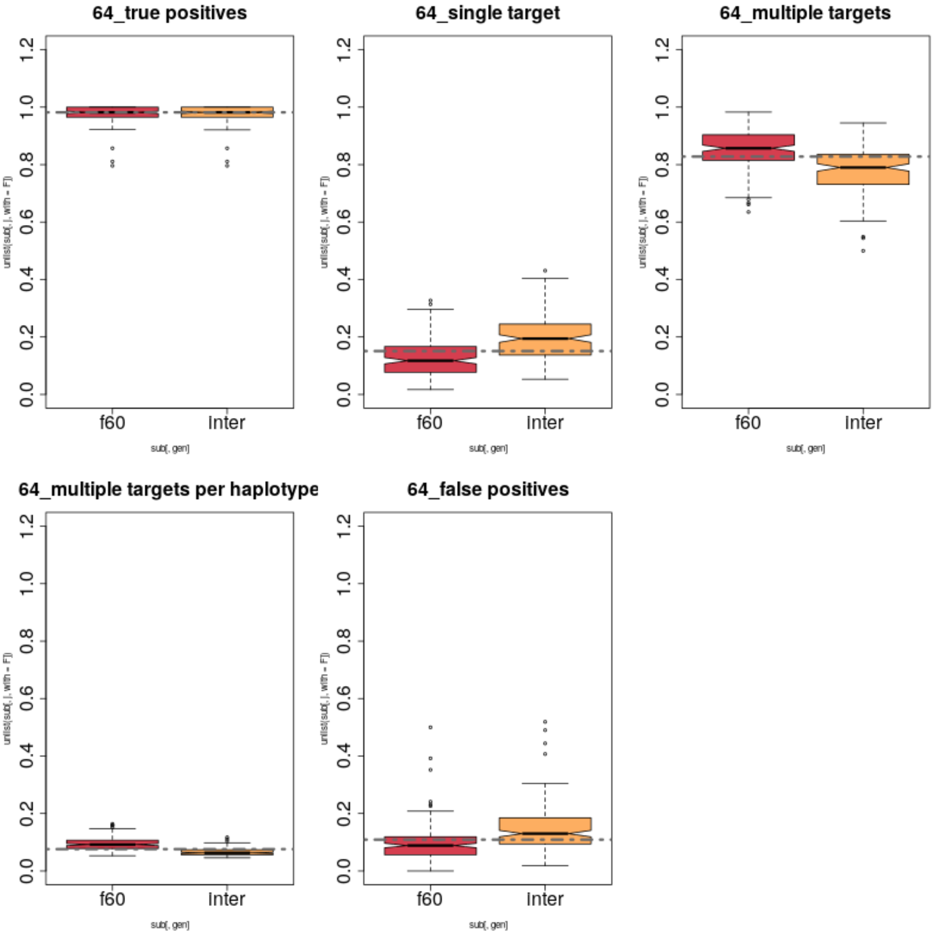

Figure 4: Haplovalidate with and without including intermediate time-points for simulations containing 64 selection targets. A: True positive rate, B: fraction of selected regions with a single selection target, C: fraction of haplotype blocks with multiple targets, D average number of selection targets on a haplotype block, E: false positive rate. Dashed line represents the overall median for each parameter.

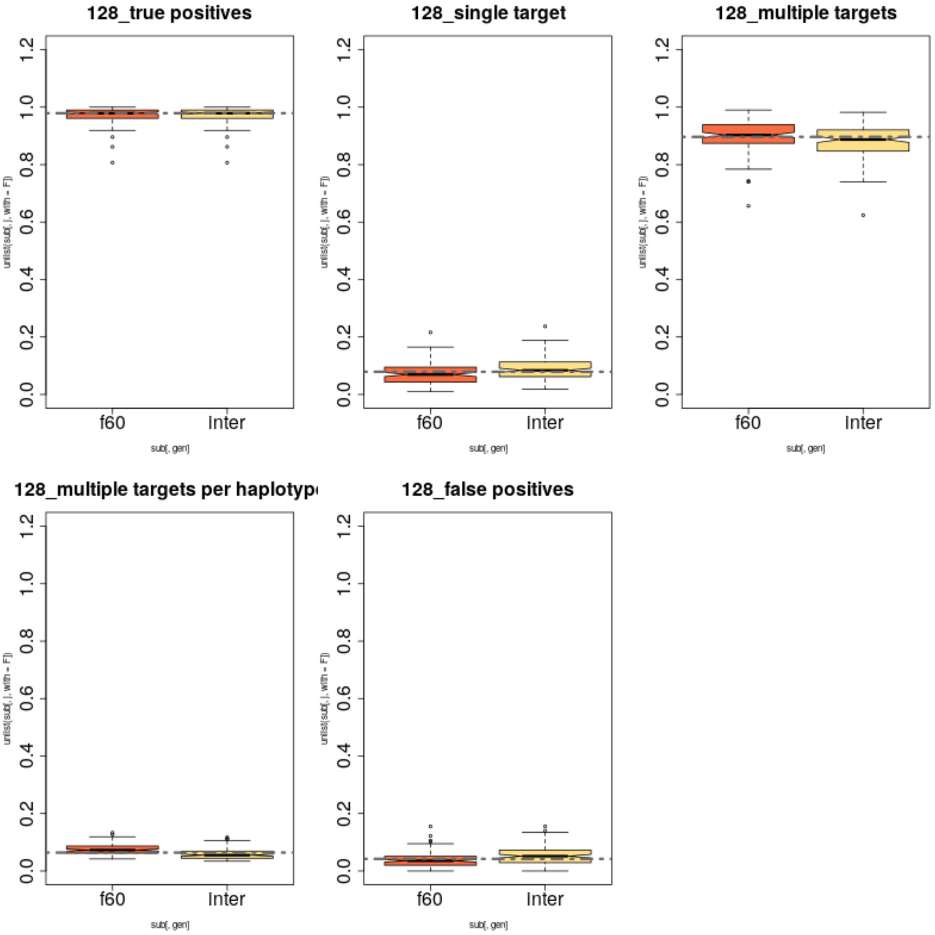

Figure 5: Haplovalidate with and without including intermediate time-points for simulations containing 128 selection targets. A: True positive rate, B: fraction of selected regions with a single selection target, C: fraction of haplotype blocks with multiple targets, D average number of selection targets on a haplotype block, E: false positive rate. Dashed line represents the overall median for each parameter.
